# The prone position in healthy pregnant women and in women with preeclampsia – a pilot study

**DOI:** 10.1186/s12884-018-2073-x

**Published:** 2018-11-16

**Authors:** Alicia T. Dennis, Liesel Hardy, Liz Leeton

**Affiliations:** 1 Departments of Pharmacology, Obstetrics and Gynaecology, and Medicine and Radiology, The University of Melbourne, Parkville, Australia; 2 Department of Anaesthesia, The Royal Women’s Hospital, Locked Bag 300, Corner Flemington Rd & Grattan St, Parkville, Victoria 3052 Australia; 3 King Edward Memorial Hospital, 374 Bagot Rd, Subiaco, WA 6008 Australia

**Keywords:** Pregnancy, Positioning, Preeclampsia, Blood pressure, Prone

## Abstract

**Background:**

The prone position is rarely used in medical settings in pregnancy. There is no published information about the prone position in women with preeclampsia. This study examined the feasibility and acceptability of the prone position in pregnant women, and the short-term effect of the prone position on blood pressure (BP) in term healthy pregnant women and in women with preeclampsia.

**Methods:**

After ethics approval, written consent and trial registration (ACTRN:12615000160538 registered 18/02/2015, date of first participant enrolled 03/03/2015), 50 healthy term pregnant women and 15 women with preeclampsia had BP, heart rate (HR), oxygen saturation (SpO2), respiratory rate (RR), fetal heart rate (FHR) and comfort levels measured in two positions: left lateral, and prone. Measurements were after five minutes rest in each position.

**Results:**

Mean ± SD age, gestation and body mass index for healthy pregnant women was 33 ± 4.1 years, 38 ± 1.0 weeks and 27 ± 3.2 kg.m^− 2^ and for women with preeclampsia was 32 ± 4.7 years, 36 ± 3.4 weeks, 31 ± 5.6 kg.m^− 2^ respectively. No clinically significant changes occurred in healthy pregnant women in the prone position. Systolic BP was reduced in the prone position in women with preeclampsia (*P* = 0.019, mean difference − 6.6 mmHg, 95% confidence interval − 11.9 to − 1.3 mmHg). 33% of women with preeclampsia experienced a 10 mmHg or greater reduction in systolic BP in the prone position. 42% of healthy pregnant women and 47% of women with preeclampsia preferred the prone position to lateral.

**Conclusions:**

This is the first study to examine the prone position in women with preeclampsia. For short periods of time the prone position is feasible and comfortable in pregnant women including those at term. The prone position may reduce systolic BP in women with preeclampsia without obvious adverse effects. Larger studies with women lying for longer periods in the prone position are required. Pregnancy should not be a contraindication to the prone position for short periods of time.

**Trial registration:**

ACTRN:12615000160538

**Electronic supplementary material:**

The online version of this article (10.1186/s12884-018-2073-x) contains supplementary material, which is available to authorized users.

## Background

The prone position is a position in which a person lies horizontally with chest down and back up. With appropriate pillows it is considered a safe position for pregnant women. The position is utilised in the allied health fields and for relaxation and massage in pregnancy. In this position uterine compression of the large abdominal vessels is almost completely alleviated with a concomitant reduction in abdominal vascular resistance and improved abdominal blood flow [1]. In non-pregnant critically ill adults, respiratory mechanics are also improved [2, 3]. Despite these advantages, the prone position is rarely used in medical settings in pregnancy and there is little information about the position in pregnancy [4]. There is no published information about the prone position in women with preeclampsia.

Preeclampsia is a hypertensive condition of human pregnancy [5]. Worldwide it affects 6.5 million young women each year and is a leading global cause of maternal mortality [6]. In high income countries is the most common reason to be admitted to an intensive care unit during pregnancy [7]. Preeclampsia is also a major cause of neonatal morbidity and mortality with an estimated 300,000–500,000 neonates dying each year as the result of their mother having preeclampsia [8]. Many neonatal deaths are the result of complications of prematurity. The maternal complications of hypertension, renal impairment, acute pulmonary oedema, systolic and diastolic cardiac failure, and intracerebral haemorrhage, are directly related to the cardiovascular system and altered haemodynamics. Long-term risks include hypertension, ischaemic heart disease and cerebrovascular disease [9].

In 2014 a new unified theory of preeclampsia has been published [10]. It proposed that the development of hypertension in preeclampsia is an adaptive response to the demands of a growing fetus and is driven by an imbalance between maternal oxygen supply to the fetus (which may be caused by inadequate blood flow to the uteroplacental unit) and fetal oxygen demands. We hypothesised that placing a woman with preeclampsia in the prone position will reduce her blood pressure by eliminating abdominal compression of blood vessels and reducing abdominal vascular resistance. This study aimed to assess feasibility and acceptability of the prone position in women in late pregnancy (healthy women and women with preeclampsia) and to determine whether blood pressure was initially reduced in the prone position in women with preeclampsia [11, 12].

## Methods

After institutional ethics approval (Royal Women’s Hospital Parkville, Australia, Human Research Ethics Approval number 14/41), written informed consent, including publication of photographs, and prospective trial registration with the Australian and New Zealand Clinical Trial Registry (ACTRN:12615000160538, https://anzctr.org.au/Trial/Registration/TrialReview.aspx?id=367699, date registered 18/02/2015, date of first participant enrolled 03/03/2015) we conducted a single centre prospective observational study at a large tertiary referral obstetric centre. Two separate groups of pregnant women were recruited. Fifty healthy term pregnant women, and 15 women with preeclampsia.

Heathy pregnant women were recruited from the antenatal clinics or from the booked elective caesarean section operating list. Inclusion criteria were any healthy term (≥ 37 weeks gestation) pregnant women defined as American Society of Anesthesiologists (ASA) Classification II, with no significant medical or surgical illness, age 18–45 years. Exclusion criteria were use of vasoactive medication including salbutamol and thyroxine, pre-existing or gestational diabetes, known cardiovascular disease including chronic disorders that may influence the cardiovascular system such as chronic renal disease, multiple pregnancy, smokers, any woman in labour or postpartum, and inability to consent to study.

Women with preeclampsia were recruited from the acute care areas of the hospital including the emergency department, day admission/assessment area, pregnancy clinics, hospital wards and birthing suite. Inclusion criteria were pregnant women aged 18–45 years meeting the criteria for preeclampsia defined by international recommendations [13, 14]. The International Society for the Study of Hypertension in Pregnancy (ISSHP) definition for preeclampsia was used. Preeclampsia was defined as hypertension developing after 20 weeks gestation and the coexistence of one or more of the following new onset conditions: proteinuria, other maternal organ dysfunction: renal insufficiency (creatinine > 90 umol/L), liver involvement (elevated transaminases and/or severe right upper quadrant or epigastric pain), neurological complications (examples include eclampsia, altered mental status, blindness, stroke, or more commonly hyperreflexia when accompanied by clonus, severe headaches when accompanied by hyperreflexia, persistent visual scotomata), haematological complications (thrombocytopenia, DIC, haemolysis) or uteroplacental dysfunction fetal growth restriction [14]. Women were defined as having severe preeclampsia if they had ongoing or recurring severe headache, eclampsia, visual field changes, nausea or vomiting, epigastric pain, severe hypertension, progressive derangements in laboratory tests (such as progressive thrombocytopenia, increasing creatinine or liver transaminases, haemolysis) or fetal growth failure or abnormal Doppler findings [13]. Exclusion criteria were any woman with pre-existing diabetes, multiple pregnancy, any woman in labour, any woman with decompensated disease (eclampsia, pulmonary oedema) or postpartum and inability to consent to study.

All participants (healthy women and women with preeclampsia) rested in the left lateral flat position on a comfortable bed in a quiet, temperature-controlled environment for five minutes before measurements. All times in positions were measured using an electronic timer on a smartphone. Blood pressure was measured non-invasively using a calibrated sphygmomanometer on the left arm recording the diastolic value as Korotkoff V according to the American Heart Association to obtain an accurate resting value [15]. An automatic blood pressure device was attached (Welch Allyn Nellcor Spot Vital Signs Monitor, Welch Allyn NY, USA 2013) and an automatic blood pressure reading was taken. If the blood pressure obtained via the automatic device are within 4 mmHg of manual blood pressure then this device was used to measure blood pressure in the prone position. Heart rate oxygen levels (using pulse oximetry) were measured automatically (Welch Allyn Nellcor Spot Vital Signs Monitor, Welch Allyn NY, USA 2013) and respiratory rate was measured by an investigator visually observing the number of breaths in 30 s and doubling this. Three blood pressure measurements were recorded, each taken after 15 s of the previous. The average of the three readings for SBP and DBP was calculated. Fetal heart rate was measured in the left lateral position using Doppler Sonicaid ultrasound device (Huntleigh Healthcare Ltd., Cardiff, UK 2014).

**Fig. 1:**
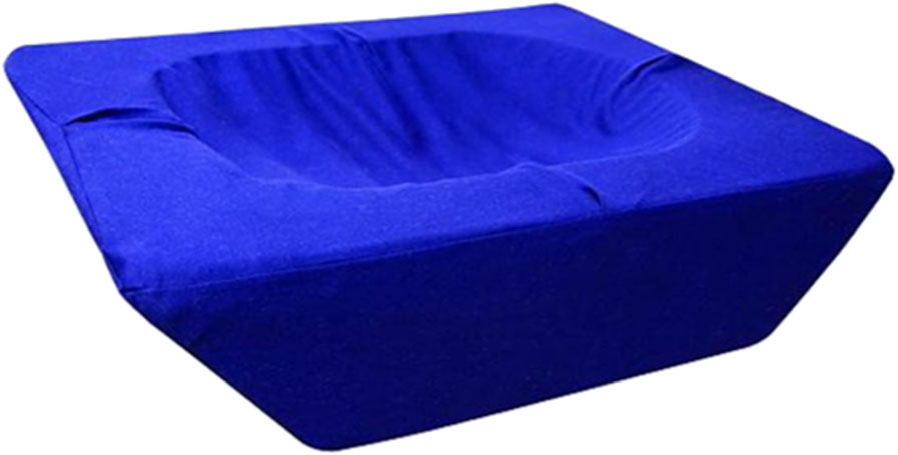
Belly Pillow®. The Belly Pillow is a specifically designed pillow for pregnant women. This image shows the larger pillow designed for women in late pregnancy. Image from https://www.bellypillow.com/content/late-pregnancy-belly-pillow Viewed 10/01/2018

**Fig. 2:**
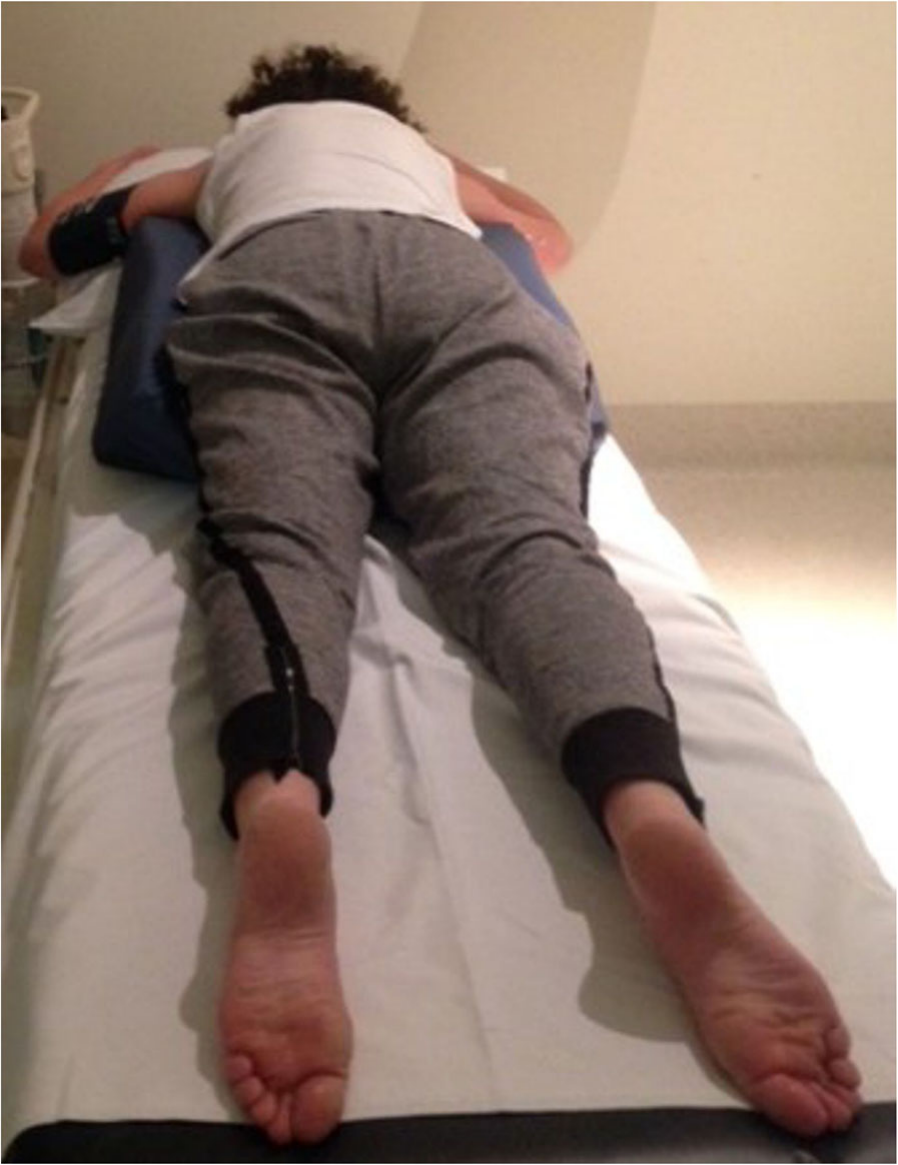
Study participant in the prone position on the BellyPillow®. This study participant is lying in the prone position on the BellyPillow®. All participants rested in this position for five minutes and then underwent study measurements

The women were then asked to position themselves prone on the BellyPillow®. This is a specially designed pillow with a centre hole that supports the gravid uterus (Figs. 1 and 2). It can be used at all gestations and for all height and weight ranges, and as women position themselves on the pillow it can accommodate all positions and ensures comfort for the woman. The BellyPillow® is used in the Royal Women’s Hospital physiotherapy department for pregnancy exercise classes. It has not been tested in a previous study. Once on the pillow in the prone position they rested for five minutes before the measurements. Blood pressure was then obtained non-invasively using a calibrated sphygmomanometer on the left arm recording the diastolic value as Korotkoff V according to the American Heart Association. Heart rate, respiratory rate and oxygen levels (using pulse oximetry) were measured. Three blood pressure measurements were recorded, each after 15 s of the previous. The average of the three readings for SBP and DBP was calculated. After the measurements were completed the women were asked to rate their comfort level in the prone position on a scale of 0 to 10 with 0 being least comfortable and 10 being most comfortable [16]. Women were then asked whether this position was more or less comfortable than the lateral position and if they had the option of resting in this position in their own time would they choose to rest this way. The participant then moved back to the left lateral position where the post-prone position fetal heart rate was measured using Doppler Sonicaid ultrasound device (Huntleigh Healthcare Ltd., Cardiff, UK 2014).

Data were analysed using SPSS Statistics Version 24 software (IBM© SPSS© Statistics Version 24 IBM Corporation 2016, Chicago, Ill). Participant characteristics, and physiological variables, were displayed as mean and standard deviation, median with interquartile ranges, or number and percentage as appropriate. Variables were compared using a two-tailed paired t test with presentation of the mean differences and 95% confidence interval. Pearson correlation coefficient with 95% CI was calculated to examine the relationship between systolic blood pressure changes and changes in heart rate. The Wilcoxon matched pairs signed rank test was used to compare comfort scores between the positions. We defined feasibility and acceptability of the prone position, using the specifically designed pillow, as there being no evidence (*p* > 0.1) of a difference in median comfort scores between the two positions. The primary outcome measurement was a change in systolic blood pressure before and after being placed in the prone position. Secondary outcomes were changes in diastolic blood pressure, maternal heart rate, oxygen saturation, respiratory rate and changes in fetal heart rate before and after being placed in the prone position. We planned a study of a continuous response variable from matched pairs of study subjects. Previously published data in our population determined that the mean and standard deviation of SBP and DBP, in healthy term pregnant women, was 119 ± 13.0 mmHg and 72 ± 11.6 mmHg respectively, and that the mean and standard deviation of SBP and DBP in women with preeclampsia was 147 ± 7.8 mmHg and DBP 93 ± 6.5 mmHg [17]. For this study, we assumed a clinically significant difference in systolic blood pressure, with the change in posture from the lateral position to the prone position, of 10 mmHg. For healthy pregnant women, if the true difference in the mean response of matched pairs was 10, we needed to study 28 subjects to be able to reject the null hypothesis that this response difference was zero with probability (power) 0.9. The Type I error probability associated with this test of this null hypothesis was 0.01 (PS Power and Sample Size Calculations Version 3.0, January 2009.

Copyright © 1997–2009 by William D. Dupont and Walton D. Plummer.) For women with preeclampsia, if the true difference in the mean response of matched pairs is 10, we needed to study 13 subjects with preeclampsia to be able to reject the null hypothesis that this response difference was zero with probability (power) 0.9. The Type I error probability associated with this test of this null hypothesis was 0.01. In order to assess feasibility and acceptability of the prone position in pregnant women we increased the sample size to a convenience sample of 50 women, and to account for possible variability in blood pressure we increased the sample size in women with preeclampsia to 15 women.

## Results

Fifty healthy pregnant women and 15 women with preeclampsia completed the study. All women found the prone position acceptable. Participant characteristics for the healthy pregnant women were mean ± SD age 33 ± 4.1 years, gestation 38 ± 1.0 weeks, height 166 ± 6.1 cm, weight 76 ± 9.8 kg and body mass index 27 ± 3.2 kg.m^− 2^. Participant characteristics for women with preeclampsia were mean ± SD age 32 ± 4.7 years, gestation 36 ± 3.4 weeks, height 164 ± 6.8 cm, weight 86 ± 22.0 kg and body mass index 31 ± 5.6 kg.m^− 2^. Most women with preeclampsia had mild disease with the systemic feature of proteinuria (urinary protein/creatinine ratio (median (IQR) lowest, highest value) 0.02 [0.05] 0.01,0.17 mmol/L). Ten women were managed without antihypertensive agents. Four women were treated with oral labetalol and one woman required oral labetalol and nifedipine. One woman had preeclampsia with severe features (severe hypertension, SBP 170 DBP 110 mmHg).

**Fig. 3:**
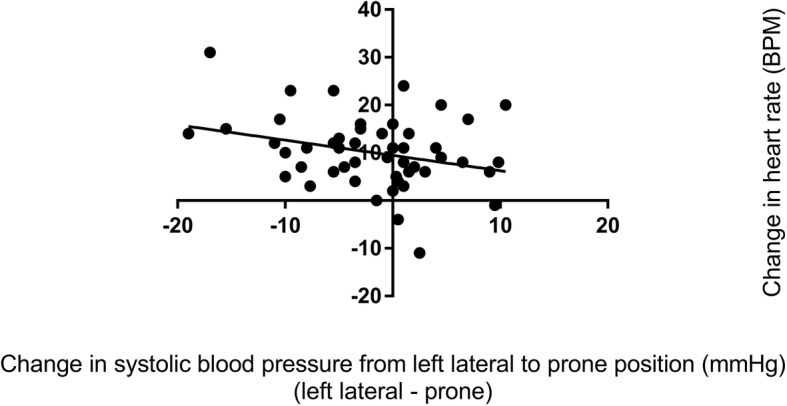
Correlation between systolic blood pressure change and heart rate change in healthy pregnant women. This figure shows that relationship between changes is systolic blood pressure and heart rate when healthy pregnant women changed from the left lateral to prone position

**Fig. 4:**
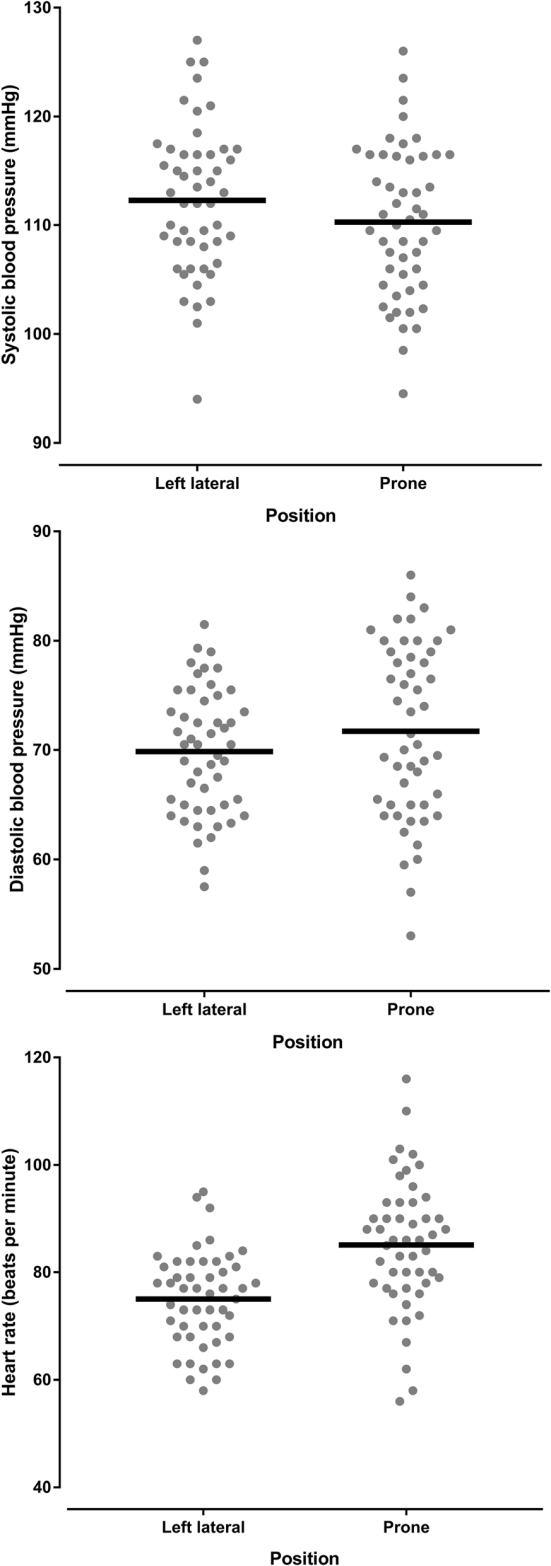
Dot plots of the systolic, diastolic and heart rate variables in healthy pregnant women in the left lateral position and the prone position. This figure shows the individual systolic, diastolic and heart rate measurements obtained from healthy pregnant women in the left lateral position and the prone position. The horizontal black lines represent the mean value

**Fig. 5:**
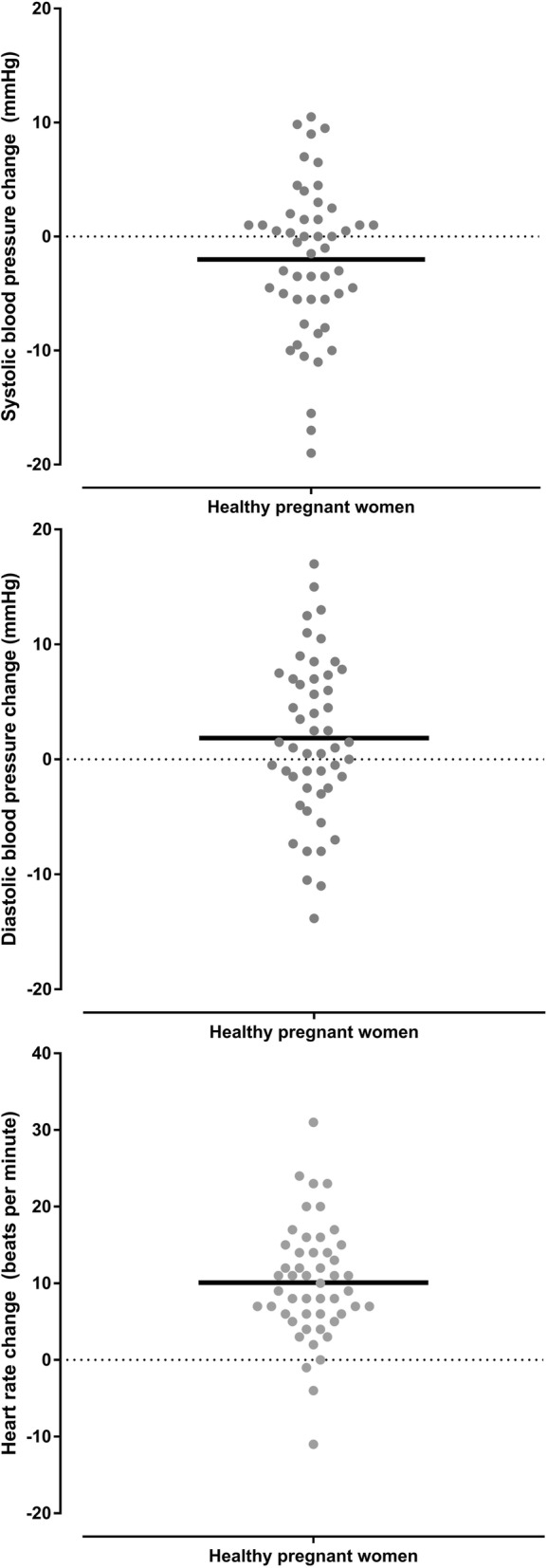
Dot plots of the changes in systolic, diastolic and heart rate variables in healthy pregnant women from the left lateral position to the prone position. This figure shows the individual systolic, diastolic and heart rate changes obtained from healthy pregnant women between the left lateral position and the prone position. The horizontal black lines represent the mean value

Haemodynamic and respiratory variables for healthy pregnant women are shown in the Table 1. Whilst there was a reduction in systolic blood pressure in the prone position compared with the left lateral position (*p* = 0.039), the mean difference was small (2 mmHg) and 95% confidence interval did not include clinically insignificant differences. There was a difference in diastolic blood pressure in the prone position (*p* = 0.064) which was clinically insignificant. There was an increase in maternal heart rate in the prone position compared with the left lateral position (*p* < 0.001 mean difference 10 BPM, 95% CI 8 to 12 BPM). There was a greater heart rate increase associated with a greater systolic blood pressure decrease (*r* = − 0.287 05% CI -0.524 to − 0.009, *P* = 0.043) meaning that a systolic blood pressure decrease (in the prone position compared to the left lateral position) was associated with a heart rate increase (in the prone position compared to the left lateral position) (Fig. 3). The changes in systolic and diastolic blood pressure and heart rate between the left lateral and prone position in healthy pregnant women are shown in Figs. 4 and 5, and Additional file 1: Figure S1. There was no difference in respiratory rate, oxygen saturation, fetal heart rate or comfort scores in the prone position compared with the left lateral position (*p* > 0.340). The prone position was preferred to the left lateral position by 21 (42%) women.

**Table 1:** Lateral and prone position in healthy pregnant women – physiological variables, comfort scores and preferred position

Variable	Position		*P* value	Mean difference	95% confidence interval
	Left lateral	Prone			
Systolic blood pressure (mmHg)	112 ± 6.8	110 ± 6.9	0.039	− 2.0	−3.9 to − 0.1
Diastolic blood pressure (mmHg)	70 ± 5.7	72 ± 8.0	0.064	1.9	− 0.1 to 3.8
Mean arterial pressure (mmHg)	84 ± 5.6	85 ± 7.2	0.521	0.6	−1.2 to 2.3
Heart rate (BPM)	75 ± 8.8	85 ± 12.2	< 0.001	10.1	8.0 to 12.2
Respiratory rate (BrPM)	17 ± 2.9	17 ± 3.0	0.347	0.4	−0.4 to 1.2
Oxygen saturation (SpO_2_%)	98 ± 0.8	98 ± 0.7	0.382	0.1	−0.2 to 0.4
Fetal heart rate (BPM)	137 ± 9.9	138 ± 9.0	0.673	0.6	−2.4 to 3.7
Comfort score	7 (6,8.125)	7 (6,8)	0.873		
Preferred position	29 (58%)	21 (42%)			

Data are mean ± SD, median (quartiles), number of women (%) as appropriate. SpO_2_ = oxygen saturation obtained by pulse oximetry, BPM = beats per minutes, BrPM = breaths per minute

**Fig. 6:**
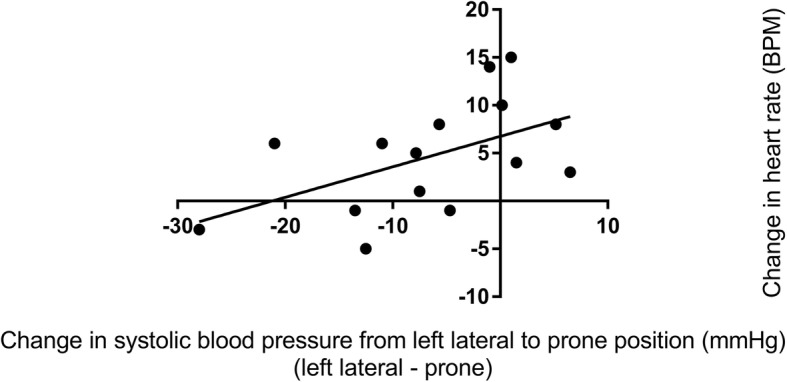
Correlation between systolic blood pressure change and heart rate change in women with preeclampsia. This figure shows that relationship between changes is systolic blood pressure and heart rate when women with preeclampsia changed from the left lateral to prone position

**Fig. 7:**
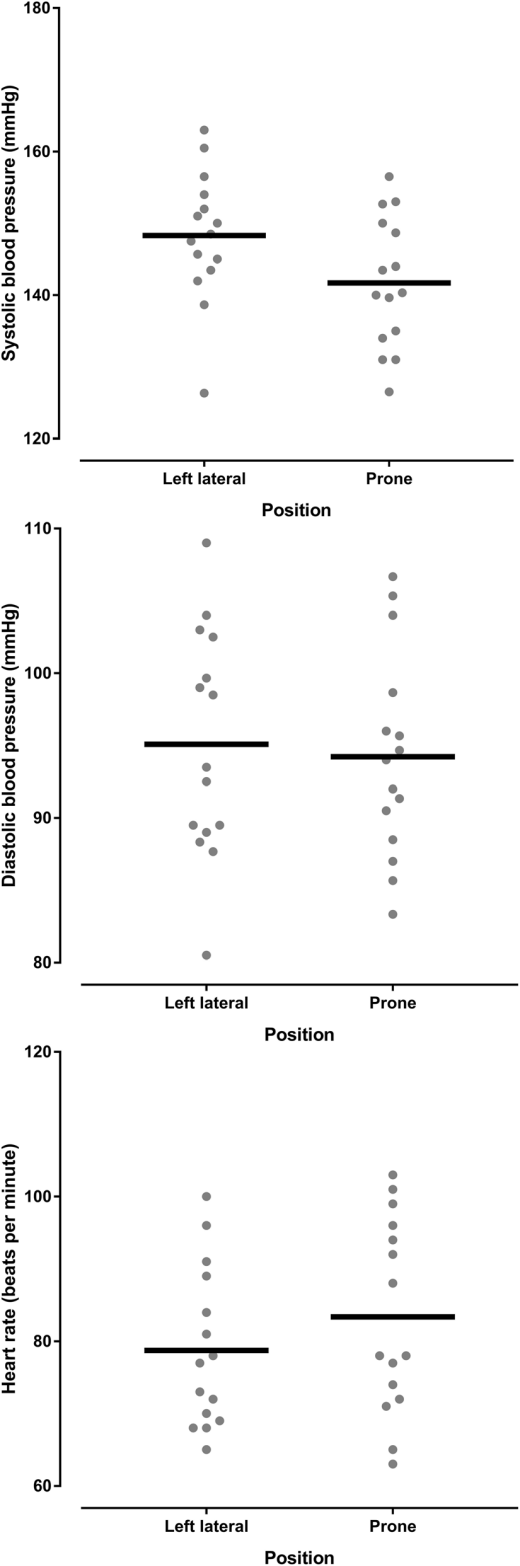
Dot plots of the systolic, diastolic and heart rate variables in women with preeclampsia in the left lateral position and the prone position. This figure shows the individual systole, diastolic and heart rate measurements obtained from women with preeclampsia in the left lateral position and the prone position. The horizontal black lines represent the mean value

**Fig. 8:**
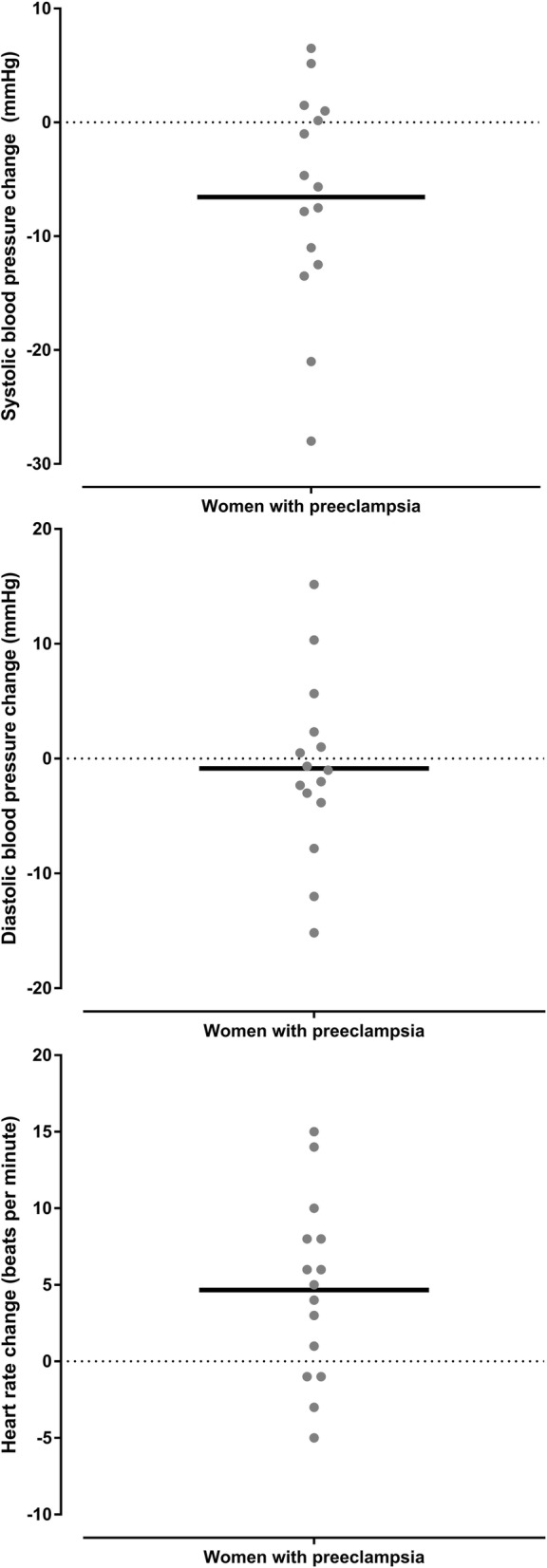
Dot plots of the changes in systolic, diastolic and heart rate variables in women with preeclampsia women from the left lateral position to the prone position. This figure shows the individual systolic, diastolic and heart rate changes obtained from women with preeclampsia between the left lateral position and the prone position. The horizontal black lines represent the mean value

**Fig. 9:**
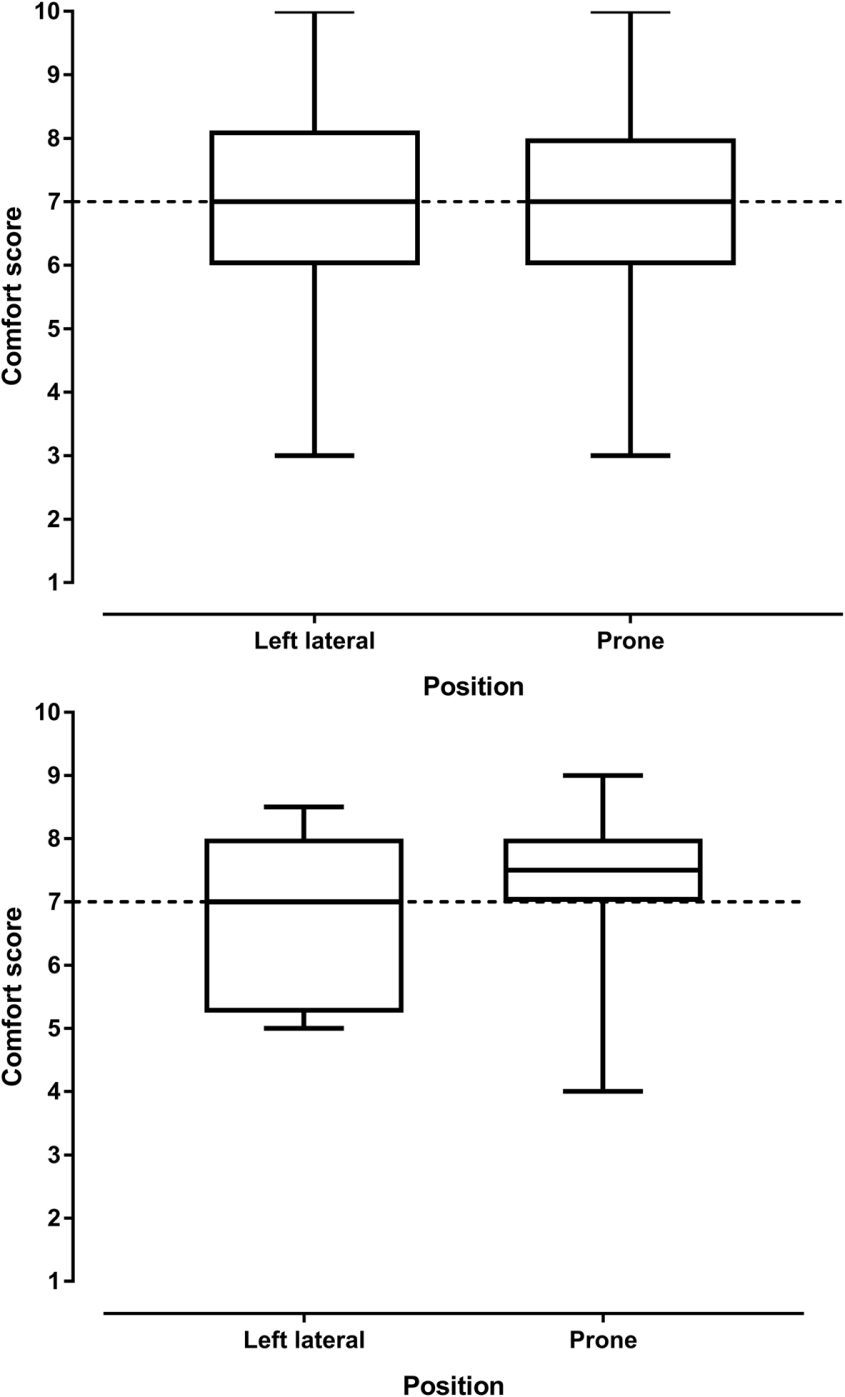
Comfort scores for healthy pregnant women and women with preeclampsia in the left lateral and prone positions. The upper figure shows comfort scores in healthy pregnant women and the lower figure shows comfort scores in women with preeclampsia

Haemodynamic and respiratory variables for women with preeclampsia are shown in the Table 2. There was a reduction in systolic blood pressure in the prone position compared with the left lateral position (*p* = 0.019). Five of the 15 (33%) women with preeclampsia experienced a reduction of 10 mmHg or more in the prone position with none experiencing a significant rise in systolic blood pressure. There was an increase in maternal heart rate in the prone position compared with the left lateral position (*p* = 0.008 mean difference 5 BPM, 95% CI 1 to 8 BPM). There was a greater heart rate increase associated with a greater systolic blood pressure increase (*r* = 0.520 95% CI 0.011 to 0.815, *P* = 0.047), meaning that a systolic blood pressure increase (in the prone position compared to the left lateral position) was associated with a heart rate increase (in the prone position compared to the left lateral position) (Fig. 6). The changes in systolic and diastolic blood pressure and heart rate between the left lateral and prone position in women with preeclampsia are shown in Figs. 7 and 8, and Additional file 1: Figure S2. There was a reduction in respiratory rate in the prone position however the difference of approximately one breath per minute was not clinically significant. There was no difference in oxygen saturation, diastolic blood pressure, fetal heart rate or comfort scores in the prone position compared with the left lateral position (*p* > 0.124). The prone position was preferred to the left lateral position by seven (47%) women. Comfort scores for healthy pregnant women and women with preeclampsia are shown in Fig. 9.

**Table 2:** Lateral and prone position in women with preeclampsia – physiological variables, comfort scores and preferred position

Variable	Position		*P* value	Mean difference	95% confidence interval
	Left lateral	Prone			
Systolic blood pressure (mmHg)	148 ± 9.0	142 ± 9.1	0.019	− 6.6	− 11.9 to − 1.3
Diastolic blood pressure (mmHg)	95 ± 7.8	94 ± 7.1	0.674	−0.9	− 5.1 to 3.4
Mean arterial pressure (mmHg)	113 ± 7.8	110 ± 7.0	0.192	−2.8	− 7.1 to 1.6
Heart rate (BPM)	79 ± 11.1	83 ± 13.4	0.008	4.7	1.4 to 7.9
Respiratory rate (BrPM)	18 ± 3.4	17 ± 2.9	0.036	−0.7	−1.4 to 0.1
Oxygen saturation (SpO_2_%)	98 ± 0.9	98 ± 0.6	0.582	0.1	−0.4 to 0.6
Fetal heart rate (BPM)	138 ± 7.4	141 ± 9.0	0.232	2.2	−1.6 to 6.0
Comfort score	7 (5.25,8)	7.5 (7,8)	0.125		
Preferred position	8 (53%)	7 (47%)			

Data are mean ± SD, median (quartiles), number of women (%) as appropriate. SpO_2_ = oxygen saturation obtained by pulse oximetry, BPM beats per minutes, BrPM breaths per minute

## Discussion

This study examines the feasibility and acceptability of the prone position in late third trimester women and investigates the association between the prone position and immediate blood pressure response in women with preeclampsia. In women with preeclampsia the prone position initially reduced systolic blood pressure by a clinically significant value of at least 10 mmHg in 33% of women. We found that the prone position is feasible and acceptable in women in late pregnancy and that the prone position was preferred to the left lateral position by at least 40% of women in the study.

This study has three important implications. The first is that placing some women with preeclampsia into the prone position initially reduces blood pressure in some women in the immediate short term. The observed blood pressure changes in women with preeclampsia are however small and limited to systolic pressure. This means that they would not be a sufficient alternative to antihypertensive therapy or change practice however these data support future studies with a design that tests the effects longer periods in the prone position. There may be particular benefit to offloading the inferior vena cava in patients with placental insufficiency and so benefit of this position in these women. In our study monitoring of maternal vital signs was possible in the prone position however external monitoring of the fetus is difficult. In both healthy pregnant women, and women with preeclampsia the prone position was associated with an increase in maternal heart rate. Further study is needed to investigate this finding and whether it is an initial baroreceptor reflex in some women.

The second implication is that approximately 40% of women in late pregnancy prefer the prone position to the lateral position and so could be offered this position, with appropriate support pillow(s), as an option during pregnancy. However it is important to note that whilst participants reported feeling comfortable in the prone position, this was for only five minutes. Without further studies it is unclear how practical this position would be and for what period this would be tolerated by most women. There may be disadvantages of lying in the prone position, such as increased back pain however no woman in our study reported such discomfort.

The third implication of this study is that the prone position can safely be used in pregnant women in late pregnancy for short periods of time. The use of a specifically designed pregnancy pillow in these settings such as intensive care units may allow the prone position to be achieved safely in critically ill pregnant women.

A limitation of this study is that it represents a preliminary study investigating the effect of the prone position in women with preeclampsia in the antenatal period. The prone position was only used in women with preeclampsia for a short period of time and a convenience sample rather than a randomized sample of pregnant women was used and these are weaknesses of the study. It is not therefore possible to determine the medium or long-term effects on blood pressure. While fetal heart rate was measured, fetal non-stress testing was not performed. Given the small number of women with preeclampsia it is unclear whether differences in blood pressure are specific to women with preeclampsia of different gestational ages, different sizes of gravid uterus and differences in body mass index.

Future studies should include a randomised controlled trial to examine the duration and frequency required in the prone position to produce a clinically significant and sustained reduction in blood pressure in women with preeclampsia.

## Conclusions

The prone position is feasible and comfortable in pregnant women including those at term. The prone position may reduce systolic blood pressure in women with preeclampsia without immediate obvious adverse effects. Randomised controlled trials are needed.

## Additional file

Additional file 1: **Figure S1.** Changes in systolic, diastolic and heart rate in each healthy pregnant woman from the left lateral position to the prone position. These figures were obtained by subtracting the variable measured in the prone position from the variable in the left lateral positions in healthy pregnant women (i.e. reductions compared to the lateral position are shown below the zero-change line). The upper figure shows changes in systolic blood pressure, the middle figure shows changes in diastolic blood pressure and the lower figure shows changes in heart rate. **Figure S2.** Changes in systolic, diastolic and heart rate in each woman with preeclampsia from the left lateral position to the prone position. These figures were obtained by subtracting the variable measured in the prone position from the variable in the left lateral positions in women with preeclampsia (i.e. reductions compared to the lateral position are shown below the zero-change line). The upper figure shows changes in systolic blood pressure, the middle figure shows changes in diastolic blood pressure and the lower figure shows changes in heart rate. (DOCX 399 kb)

## Abbreviations

ASA: American Society of Anesthesiology
BP: Blood pressure
BPM: Beats per minute
CI: Confidence interval
DBP: Diastolic blood pressure
FHR: Fetal heart rate
HP: Healthy pregnant
RR: Respiratory rate
SBP: Systolic blood pressure
SD: Standard deviation
SpO_2_: Oxygen saturation
