# Supplementary figures and images for: The prone position in healthy pregnant women and in women with preeclampsia – a pilot study
Source: BMC Pregnancy Childbirth. 2018 Nov 16;18:445. doi: 10.1186/s12884-018-2073-x (PMC6240306; doi:10.1186/s12884-018-2073-x)

**Figure S1**
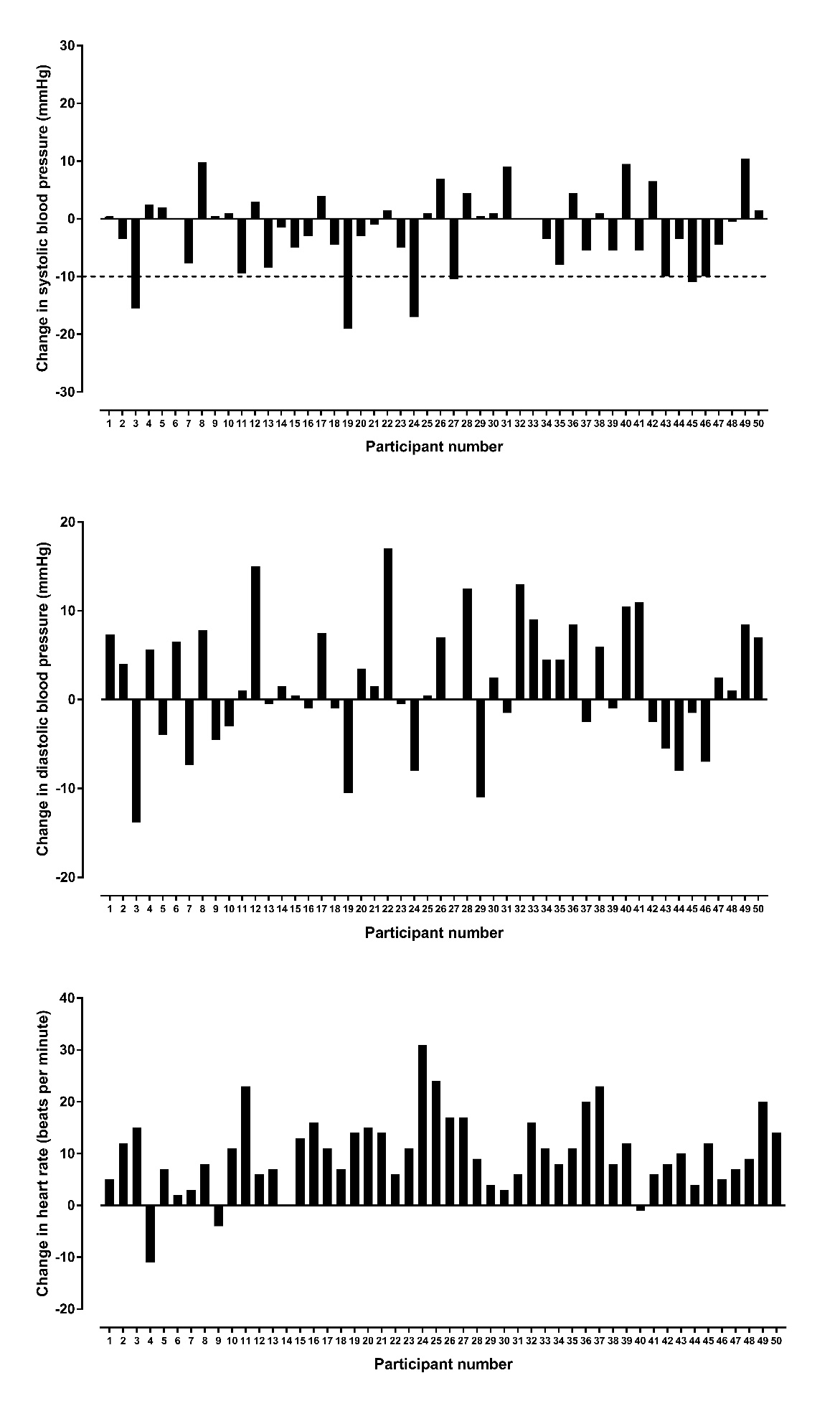


**Figure S2
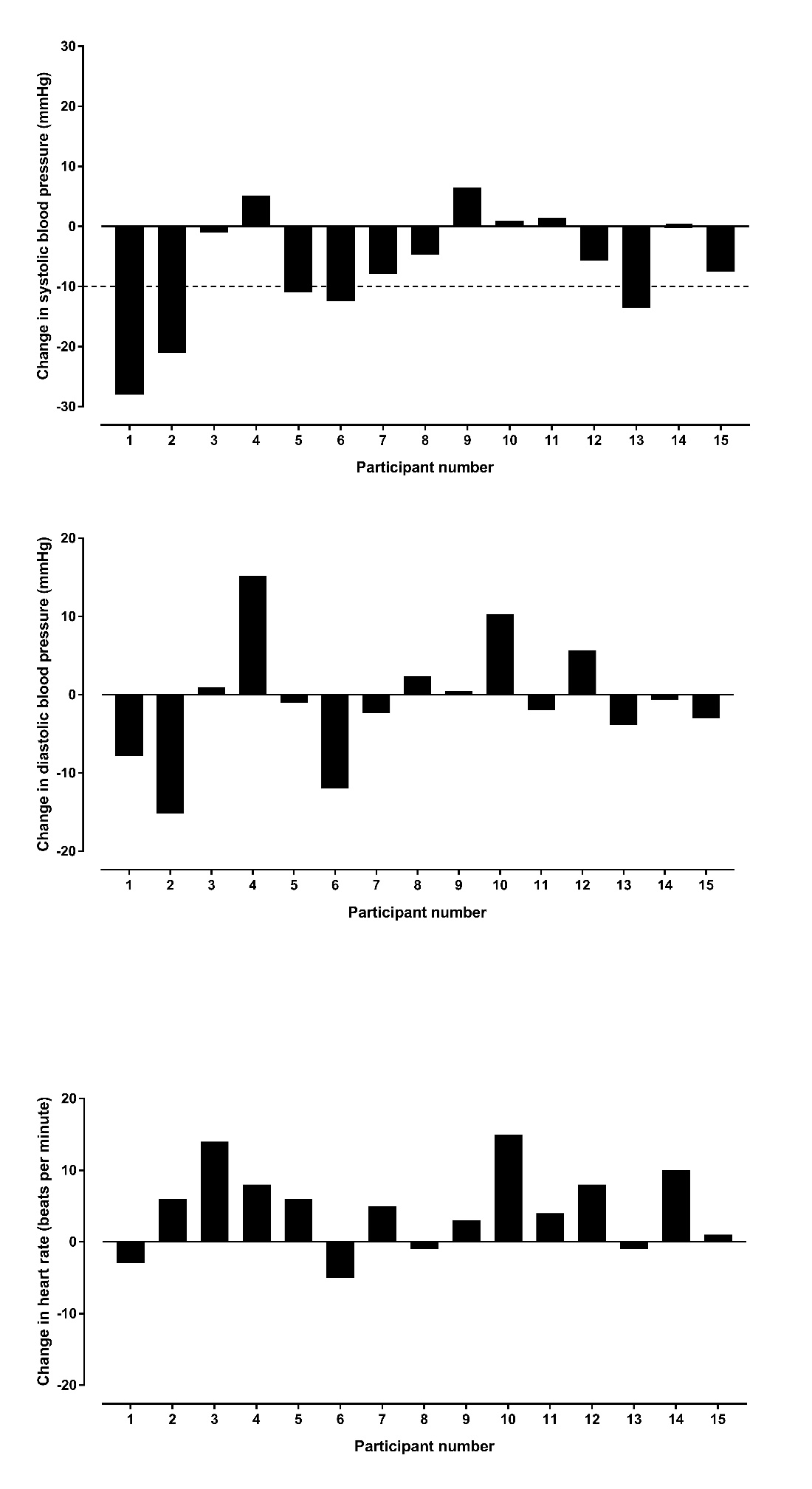
**

Supplement: Supplementary file 1 — Figure S1. Changes in systolic, diastolic and heart rate in each healthy pregnant woman from the left lateral position to the prone position. These figures were obtained by subtracting the variable measured in the prone position from the variable in the left lateral positions in healthy pregnant women (i.e. reductions compared to the lateral position are shown below the zero-change line). The upper figure shows changes in systolic blood pressure, the middle figure shows changes in diastolic blood pressure and the lower figure shows changes in heart rate. Figure S2. Changes in systolic, diastolic and heart rate in each woman with preeclampsia from the left lateral position to the prone position. These figures were obtained by subtracting the variable measured in the prone position from the variable in the left lateral positions in women with preeclampsia (i.e. reductions compared to the lateral position are shown below the zero-change line). The upper figure shows changes in systolic blood pressure, the middle figure shows changes in diastolic blood pressure and the lower figure shows changes in heart rate. (DOCX 399 kb) [file 12884_2018_2073_MOESM1_ESM.docx]
